# CO_2_-Induced Foaming and Gelation for the Fabrication of Macroporous Alginate Aerogel Scaffolds

**DOI:** 10.3390/gels12010017

**Published:** 2025-12-24

**Authors:** Natalia Menshutina, Eldar Golubev, Andrey Abramov, Pavel Tsygankov

**Affiliations:** Department of Chemical and Pharmaceutical Engineering, Mendeleev University of Chemical Technology of Russia, Miusskaya pl. 9, 125047 Moscow, Russia; chemcom@muctr.ru (N.M.); eldgol01@gmail.com (E.G.); abramovandrey516@gmail.com (A.A.)

**Keywords:** alginate aerogel, supercritical CO_2_ drying, hierarchical porosity, macroporous scaffold, tissue engineering, Peclet number, Deborah number

## Abstract

Alginate aerogels are attractive candidates for biomedical scaffolds because they combine high mesoporosity with biocompatibility and can be processed into open, interconnected macroporous networks suitable for tissue engineering. Here, we systematically investigate how CO_2_-induced foaming parameters govern the hierarchical pore structure of alginate aerogels produced by subsequent supercritical CO_2_ drying. Sodium alginate–CaCO_3_ suspensions are foamed in a CO_2_ atmosphere at 50 or 100 bar, depressurization rates of 50 or 0.05 bar·s^−1^, temperatures of 5 or 25 °C, and, optionally, under pulsed pressure or with Pluronic F-68 as a surfactant. The resulting gels are dried using supercritical CO_2_ and characterized by micro-computed tomography and N_2_ sorption. High pressure combined with slow depressurization (100 bar, 0.05 bar·s^−1^) yields a homogeneous macroporous network with pores predominantly in the 200–500 µm range and a mesoporous texture with 15–35 nm pores, whereas fast depressurization promotes bubble coalescence and the appearance of large (>2100 µm) macropores and a broader mesopore distribution. Lowering the temperature, applying pulsed pressure, and adding surfactant enable further tuning of macropore size and connectivity with a limited impact on mesoporosity. Interpretation in terms of Peclet and Deborah numbers links processing conditions to non-equilibrium mass transfer and gel viscoelasticity, providing a physically grounded map for designing hierarchically porous alginate aerogel scaffolds for biomedical applications.

## 1. Introduction

Biopolymer-based aerogels (alginate, chitosan, gelatin, collagen, etc.) are used in many fields [1,2,3]. Owing to the combination of high sorption capacity, low density, high specific surface area, biocompatibility, and elasticity, biopolymer aerogels are in demand in biomedicine, cosmetology, and the food industry.

Particular interest is focused on the use of biopolymer-based aerogels as high-performance matrices for the growth of cells and tissues. Aerogels, due to their properties, have already proven effective as matrices for the growth of stem cells and for cartilage and bone tissue regeneration [4]. However, a promising direction is the development of methods for forming a hierarchical porous structure that would integrate a macroporous network into the mesoporous structure of biopolymer aerogels. This is necessary, on the one hand, to provide efficient delivery of nutrients and oxygen and removal of metabolites, and, on the other hand, to maintain a high specific surface area for adsorption of active molecules and growth-stimulating factors [5,6]. According to the literature, optimal pore sizes in highly porous scaffolds strongly depend on the cell phenotype and the target tissue, reflecting differences in adhesion, migration, and vascularization mechanisms. For bone tissue formation (osteogenesis), pore sizes ≥ 300 µm are recommended; for chondrocytes, 100–200 µm (sometimes 300–400 µm in polycaprolactone-based scaffolds) are effective. Efficient fibroblast adhesion to the matrix is reported for ~40–150 µm; for angiogenesis, pores of ~35–100 µm are recommended [7,8,9]. The presence of interconnected pores in the 100–500 µm range creates conditions for high proliferative activity of cells into the internal volume of a porous scaffold.

The methods for generating macroporosity in biopolymer materials described in the literature can be classified into approaches using “hard” templates (particles) [10,11,12], “soft” templates (emulsions and foams stabilized by surfactants) [13,14,15], and template-free approaches based on phase separation or gas generation [9,16]. Template-free methods include cryogelation, thermo-induced phase separation, and gas foaming. Gas foaming, including processes using pressurized CO_2_, is a promising approach for obtaining open, interconnected macroporosity in hydrogels and biopolymer-based aerogels [17].

Sodium alginate is one of the most widely used biopolymers investigated and applied in the field of tissue engineering. Its main advantages include low cost, high biocompatibility, support of cell proliferation, and the absence of toxicity. The geometry of sodium-alginate–based materials is largely determined by the gelation mechanism, which in turn depends on the composition of the gel-forming formulation. When gelation occurs in a crosslinking-agent solution for surfactant-modified formulations, diffusion of Ca^2+^ ions produces a concentration gradient of the crosslinker from the surface toward the core of the part. This gradient is accompanied by pronounced linear shrinkage and edge thinning. In contrast, gelation in a CO_2_ environment provides a more uniform distribution of Ca^2+^ throughout the volume. This promotes gradual crosslinking of the alginate chains and a more even redistribution of internal stresses, thereby reducing shrinkage and better preserving the part’s geometric dimensions and shape.

The key parameters of CO_2_-based foaming that determine the macroporous structure of biopolymer materials include temperature, pressure, process duration, and depressurization rate. These parameters govern the nucleation of gas bubbles, their growth and coalescence, and, consequently, the pore size, their distribution, and interconnectivity [4,18,19]. Reference [20] reported a method for producing bimodal porous scaffolds via foaming in a CO_2_ environment, which enabled colonization and osteogenic differentiation of stem cells by combining small and large tubular pores. Study [21] introduced innovative scaffolds obtained by a one-step CO_2_ foaming process that demonstrated successful adhesion and growth of mesenchymal stem cells and human umbilical-cord-derived cells. Work [22] showed the effectiveness of CO_2_-based foaming for producing reinforced composite foams, addressing the insufficient mechanical durability of biodegradable scaffolds. Investigation [23] demonstrated the feasibility of creating highly porous, mechanically robust matrices from aqueous sodium-alginate solutions with added glycerol by foaming in CO_2_ under high pressure (sub- and supercritical regimes); porosity of the structures increased with gas pressure.

This work presents a study of the foaming process for sodium alginate-based biopolymer materials in a CO_2_ environment. Structural characterization of the obtained materials was used to identify specific features of the foaming process and to elucidate how process parameters and additional pore-forming components affect the formation of the macroporous structure.

## 2. Results and Discussion

### 2.1. Mechanism of CO_2_-Induced Gelation and Foaming in the Alginate–CaCO_3_ System

**Figure 1:**
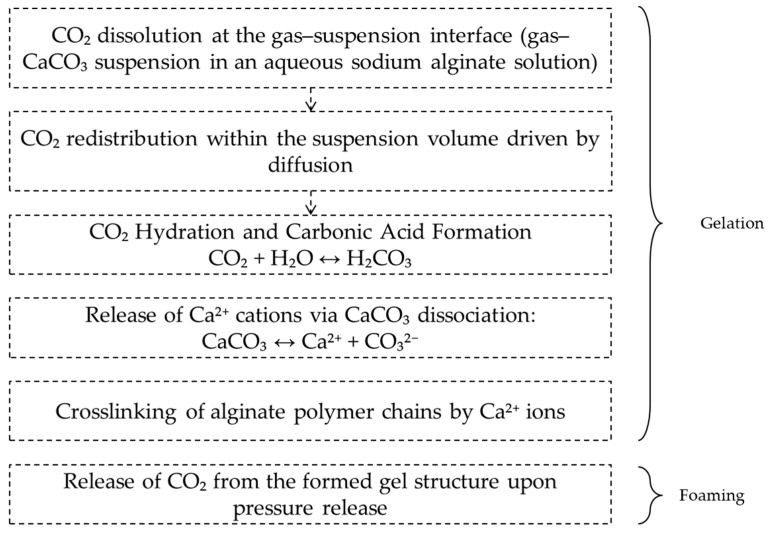
Schematic representation of CO_2_-Induced Gelation and Foaming in the Alginate–CaCO_3_ System.

The formation mechanism of the macroporous structure in sodium alginate–based gels is governed by the combined effects of mass transfer, acid–base equilibrium, ionic crosslinking, and system supersaturation. It proceeds through two consecutive stages: gelation and foaming. Figure 1 presents a schematic illustration of macroporous structure formation under pressurized CO_2_ in the “sodium alginate–calcium carbonate” system.

At the first stage, carbon dioxide dissolves in the sodium alginate–calcium carbonate suspension. CO_2_ then diffuses throughout the suspension volume. Increasing the pressure in the system enhances the solubility of carbon dioxide in accordance with Henry’s law (Equation (1)):

(1) $$C_{{CO}_{2}}=k\cdot P_{{CO}_{2}},$$

Thus, an increase in system pressure leads to an increase in the concentration of carbon dioxide in the aqueous suspension [24,25]. This results in a shift of the acid–base equilibrium associated with the hydration of CO_2_ and the dissociation of carbonic acid H_2_CO_3_ in the bulk of the material (Equation (2)):

(2) $${CO}_{2}+H_{2}O↔H_{2}CO_{3}↔HCO_{3}^{-}+H^{+}↔{CO}_{3}^{2-}+{2H}^{+}$$

The formation of carbonic acid lowers the pH, which, in turn, initiates the dissolution of CaCO_3_ and the release of Ca^2+^ ions that crosslink sodium alginate. Chemical crosslinking is accompanied by an increase in the storage modulus G′, an increase in the relaxation time λ, and the formation of a solid gel before depressurization begins [26]. The pressure in the system determines two main parameters: the total amount of dissolved CO_2_ that can transition into the gas phase during depressurization, and the stiffness of the solid network, which, in turn, depends on the fraction of dissociated Ca^2+^ ions and the degree of alginate crosslinking. The higher the pressure, the higher the concentration of dissolved CO_2_ and, consequently, the lower the pH, which accelerates gelation.

At the second stage of macropore formation, the dissolved carbon dioxide is released from the formed alginate gel. Depressurization leads to supersaturation of the gel with CO_2_, triggering nucleation and growth of gas bubbles. The condition for bubble stability is that its radius exceeds the critical value (Equation (3)) [27]:

(3) $$r_{cr}=\frac{2\gamma }{∆P},$$

where $r_{cr}$ is the critical radius (m); γ is the interfacial tension (N/m); and ΔP is the local pressure drop (Pa).

Bubbles whose radius exceeds the critical value grow due to diffusive CO_2_ supply and mechanical expansion. The increasing gas volume ruptures the gel network, forming cavities within it. The competition among nucleation, growth, and coalescence of bubbles, occurring simultaneously with gel stiffening due to ongoing ionic crosslinking, leads to the formation of a macroporous structure whose characteristics are determined by the technological parameters of the process, i.e., pressure and depressurization rate. In this work, we focus on the effect of these parameters on the macro- and mesoporous structure of alginate aerogels.

### 2.2. Effect of Pressure and Depressurization Rate

To study the effect of pressure and depressurization rate on the structure of alginate-based materials, experiments were carried out at pressures of 50 and 100 bar and depressurization rates of 50 and 0.05 bar/s. The preparation of the sodium alginate–calcium carbonate suspension and the gelation and foaming procedures are described in Section 4.2.

**Figure 2:**
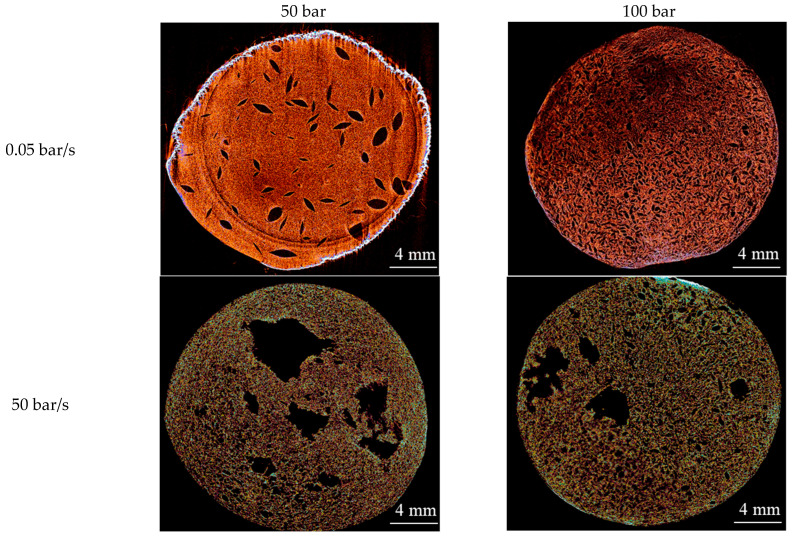
Micro-CT images of alginate aerogels obtained at different process parameters (pressure 50 or 100 bar; depressurization rate 0.05 or 50 bar/s).

The macroporous structure of the obtained materials was investigated by micro-computed tomography (micro-CT). Micro-CT images of the samples are shown in Figure 2.

Micro-CT results revealed that the depressurization rate has the strongest effect on the macroporous structure. At a high depressurization rate (50 bar/s), large pores are formed in the materials at both pressure levels (50 and 100 bar). At a low depressurization rate (0.05 bar/s), the structural pattern changes markedly, and large pores are absent. Increasing the pressure from 50 to 100 bar at a fixed low depressurization rate additionally narrows the pore size and its distribution, which may indicate an increase in nucleation site density and an increase in the stiffness of the gel network due to more complete ionic crosslinking at higher dissolved CO_2_ concentrations.

**Figure 3:**
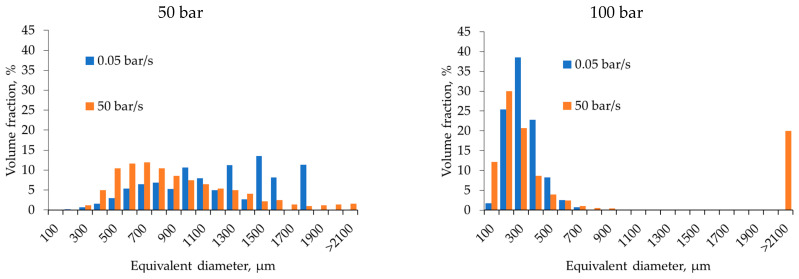
Macropore size distributions obtained from micro-CT data for alginate aerogels produced at different process parameters.

To quantify the macroporous structure, pore size distributions were calculated based on micro-CT data (Figure 3).

Analysis of the equivalent diameter distributions quantitatively confirms the trends observed in the micro-CT images. Increasing the depressurization rate from 0.05 to 50 bar/s leads to a broader pore size range and a higher fraction of large pores. The pore size distributions become asymmetric with equivalent pore diameters exceeding 1500 µm. This indicates a highly non-equilibrium degassing regime with pronounced gas bubble coalescence. At slow depressurization and an initial pressure of 50 bar, the distribution narrows significantly, with most pores in the intermediate size range (100–700 µm). Notably, increasing the pressure from 50 to 100 bar at the low depressurization rate further shifts the distribution maximum toward smaller diameters (200–500 µm) and reduces dispersion, indicating the formation of a finer and more homogeneous structure. Thus, the diagrams demonstrate the combined effects of two factors: the depressurization rate primarily determines the width of the pore size distribution and the presence of large pores, whereas higher initial pressure at slow depressurization promotes more uniform pore sizes and a narrower distribution.

**Figure 4:**
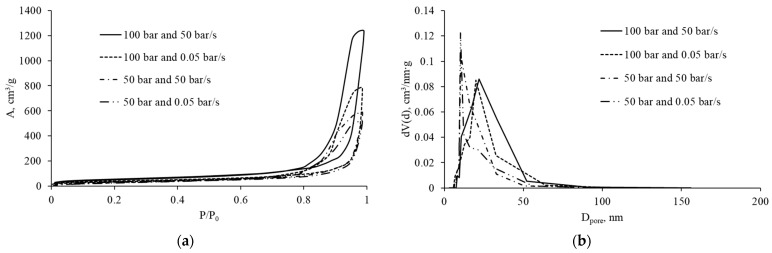
(**a**) Nitrogen adsorption–desorption isotherms and (**b**) mesopore size distributions for alginate aerogels obtained at different pressures and depressurization rates.

The mesoporous structure was investigated by low-temperature nitrogen adsorption–desorption. Figure 4 shows the nitrogen sorption isotherms and mesopore size distributions for the obtained materials.

The results demonstrate that the materials possess a mesoporous structure. The isotherms are characteristic of reversible adsorption on mesoporous materials via a polymolecular adsorption mechanism. The presence of hysteresis loops indicates capillary condensation. Table 1 summarizes the specific surface area and mesopore volume, calculated using the Brunauer–Emmett–Teller (BET) and Barrett–Joyner–Halenda (BJH) methods, respectively.

These results show that samples prepared at higher initial pressure exhibit higher specific surface area and mesopore volume. This is likely related to the formation of a more robust and dense structure at the gelation stage. It is also noteworthy that increasing the depressurization rate leads to a decrease in mesopore volume, which can be associated with gas coalescence during depressurization and, consequently, rupture of the mesoporous network and formation of macropores. This conclusion is consistent with the micro-CT analysis of the macroporous structure.

Based on the combined micro-CT, pore size distribution, and nitrogen sorption data, we can conclude that the 50–100 bar/50 bar/s regimes correspond to non-equilibrium foaming with extensive coalescence and formation of a coarse, spatially heterogeneous cellular structure, whereas regimes with a depressurization rate of 0.05 bar/s, especially at 100 bar, yield more homogeneous macroporous structures.

These findings can be generalized by directly linking the Peclet and Deborah numbers to the technological parameters of the foaming process: saturation pressure P_sat_, depressurization rate $\frac{dP}{dt}$, sample size L, and the physico-chemical properties of the gel (CO_2_ diffusion coefficient D and structural relaxation time λ).

The Peclet number describes the relationship between the rate of change of the thermodynamic field and the rate of homogenization of the dissolved CO_2_ concentration across the gel thickness. In classical transport theory, the Peclet number is defined as the ratio of diffusion time $t_{diff}=\frac{L^{2}}{D}$ to process time $t_{procc}$ and thus characterizes the degree of non-equilibrium of mass and heat transfer phenomena [28]. In the context of alginate gel foaming under CO_2_ pressure, the characteristic process time can be taken as the depressurization time tdep=ΔPdPdt, determined by the pressure drop ΔP and the depressurization rate $\frac{dP}{dt}$. In this case, the Peclet number takes the form (Equation (4)):

(4) Pe=tdifftdep=L2DdPdtΔP

The dimensionless Pe depends on the diffusion coefficient, depressurization rate, and process pressure [29,30,31]. The Péclet number describes the ratio between the time required to establish an equilibrium concentration of dissolved CO_2_ and the characteristic depressurization time, and thus quantifies the degree of non-equilibrium in the concentration field. At high Pe, the process proceeds in a strongly non-equilibrium regime, accompanied by pronounced bubble coalescence and the formation of a broad macropore size distribution, whereas at Pe ≲ 1 a quasi-steady regime is established, resulting in a narrow pore size distribution. Increasing the depressurization rate from 0.05 to 50 bar/s at fixed pressure, sample geometry, and diffusion coefficient leads to higher Pe. In this situation, the time of external condition change is much shorter than the time required for diffusive homogenization of the gas concentration throughout the material. This corresponds to the development of strong radial supersaturation gradients, formation of cavities, and broad macropore distributions, consistent with theoretical calculations for diffusion-induced bubble growth in viscoelastic polymers [31,32]. Conversely, at a low depressurization rate of 0.05 bar/s, the Peclet number decreases, the CO_2_ concentration gradient within the material becomes less pronounced, supersaturation becomes more uniform, and a homogeneous macroporous structure is formed.

The Deborah number [33] describes the relationship between the characteristic material relaxation time and the time scale of external loading. In polymer rheology, it is written as De=λtprocc, where λ is the stress relaxation time and τ is the characteristic deformation time. In the context of alginate gel foaming, τ corresponds to the depressurization time tdep=ΔPdPdt. Thus, the Deborah number can be expressed as (Equation (5)):

(5) De=λtdep=λdPdtΔP

De is determined by the processing parameters and the stiffness of the polymer network [34]. The Deborah number represents the ratio between the relaxation time of the alginate network and the characteristic depressurization time, and thus determines whether the matrix behaves as an elastic solid (De ≫ 1) or as a viscous liquid (De ≪ 1) during bubble growth. The stiffness of the polymer network, and hence λ, depends on the degree of crosslinking, which in turn depends on the concentration of dissociated calcium cations [35,36]. In the system under consideration, λ is primarily determined by the saturation pressure and the holding time at that pressure. At 50 bar a relatively “soft” network with a shorter relaxation time is formed, whereas at 100 bar the greater acidification and enhanced ionic crosslinking lead to a stiffer gel and higher λ, in accordance with general trends in polymer relaxation behavior. Combined with a high depressurization rate (50 bar/s), this yields high De values. In this case, the pressure changes faster than the gel can relax, and bubble growth occurs in a regime where elasticity dominates, resulting in network rupture, coalescence, and formation of large macropores, analogous to high-De regimes in polymer melt foaming models [37]. At a depressurization rate of 0.05 bar/s, the Deborah number shifts to moderate values. The process time is comparable to or exceeds λ, stresses in the network have time to redistribute, pore walls do not break, and pore sizes are fixed at the early stages of growth. Thus, the combination of high saturation pressure, which yields a large number of nucleation sites and sufficient gel stiffness, with a low depressurization rate corresponds to a (Pe, De) region that provides a macro- and mesoporous structure optimal for tissue engineering and efficient heat and mass transfer in alginate aerogels.

Consequently, at a saturation pressure of 100 bar the maximum CO_2_ solubility is achieved, leading to a significant pH shift to the acidic region. At a depressurization rate of 0.05 bar/s, the depressurization time is comparable to or exceeds the gel relaxation time; pressure and concentration gradients are small, the critical nucleus radius remains relatively large, and coalescence is limited. This regime yields a macroporous structure with pore sizes in the ~200–500 µm range and a mesoporous structure with pores of ~20–35 nm, both with high spatial homogeneity.

For cell scaffolds, this provides three important effects. First, a narrow macropore size distribution ensures reproducible proliferation and convective exchange of medium, oxygen, and metabolites. Second, the mesoporous structure enhances mechanical stability and provides a controlled specific surface area for adsorption of APIs and extracellular matrix proteins, which improves proliferation while maintaining diffusive permeability. Third, the absence of large pores (>2100 µm) leads to a more uniform stress distribution in the solid network, reducing the risk of deformation during sterilization, hydration, and long-term culture.

However, depending on the cell type, it is necessary to investigate the possibility of further tuning the hierarchical porosity by changing process parameters or introducing additional agents.

### 2.3. Effect of Process Temperature, Pulsed Pressure Changes, and Surfactant Addition

A series of additional experiments was carried out to expand the range of macropore sizes and to further tune the structure. The following approaches were investigated:

1. Lowering the process temperature. Reducing the foaming temperature increases CO_2_ solubility in the aqueous system, increases the viscosity of the dispersion medium, and slows diffusion. It is expected to further narrow the macropore size distribution and shift the average mesopore size toward smaller diameters due to more “complete” crosslinking during prolonged residence in the low-pH region.
2. Pulsed pressure variation during foaming. Pulsed changes in pressure can promote the formation of elongated anisotropic pores. Such anisotropic pores are attractive for guided growth of nerve, muscle, or endothelial structures.
3. Addition of a surfactant (SAA). Surfactant addition can affect the critical nucleus radius by lowering interfacial tension and influence the stability of thin walls between forming pores. In addition, low concentrations of biocompatible surfactants can increase the density of nucleation sites.

#### 2.3.1. Effect of Process Temperature

**Figure 5:**
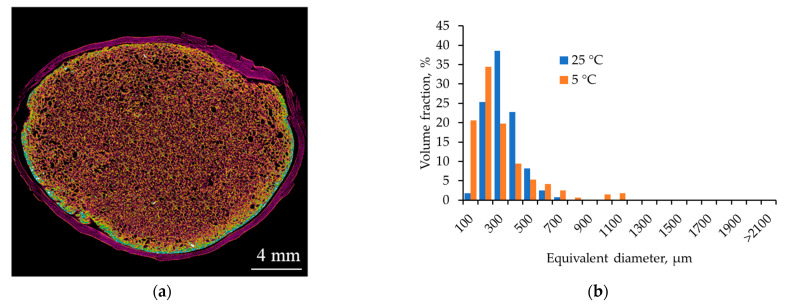

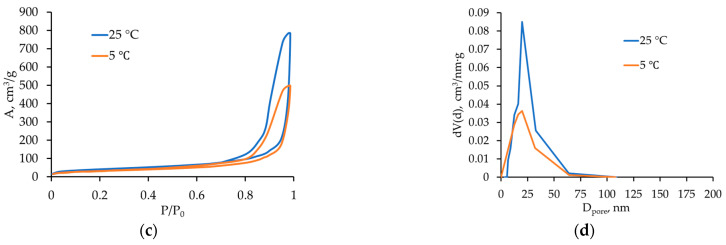
Micro-CT images and nitrogen sorption results for alginate aerogels (saturation pressure 100 bar; depressurization rate 0.05 bar/s; temperature 25 and 5 °C): (**a**,**b**) micro-CT and pore size distribution; (**c**,**d**) nitrogen adsorption–desorption isotherm and mesopore size distribution.

Figure 5 shows the micro-CT and nitrogen sorption results for alginate aerogels produced at a saturation pressure of 100 bar and a depressurization rate of 0.05 bar/s at 5 °C. The pore size distribution was calculated from the micro-CT data.

The micro-CT image shows that lowering the temperature promotes the formation of a larger number of fine pores. The pores are uniformly distributed throughout the volume; no pronounced large channels or cavities are formed.

The macropore size distribution is shifted toward smaller equivalent diameters compared with the sample obtained at 25 °C. Most pores are in the 100–300 µm range, the fraction of 400–600 µm pores is noticeably lower, and the contribution of pores larger than 800–1000 µm is minimal. This behavior is consistent with the assumption that lowering the temperature increases the number of nucleation sites (due to increased CO_2_ solubility and deeper acidification) while simultaneously increasing the resistance of the gel network to pore coarsening.

According to nitrogen sorption data, lowering the temperature at fixed pressure and depressurization rate leads to a moderate decrease in the adsorbed volume at high relative pressures and a narrowing of the hysteresis loop compared with the 25 °C sample. The adsorption isotherm remains typical of type IV mesoporous materials, while the maximum in the mesopore size distribution shifts toward slightly smaller diameters (15–25 nm) with a narrower distribution band. This is reflected in a decrease in specific surface area and mesopore volume compared with the regime of 100 bar, 0.05 bar/s, 25 °C.

Lowering the temperature to 5 °C at fixed pressure and depressurization rate simultaneously decreases the CO_2_ diffusion coefficient in the gel and increases the relaxation time λ (due to higher viscoelasticity and more extensive ionic crosslinking resulting from higher CO_2_ solubility and deeper acidification). This implies an increase in both Pe and De: diffusive fluxes become slower but remain comparable to the depressurization time, and the gel network retains its ability to redistribute stresses without wall rupture. CO_2_ supersaturation manifests as more numerous nucleation sites, while the increased network stiffness limits the growth of individual pores, giving rise to a narrower macropore size distribution. Thus, the regime of 100 bar, 0.05 bar/s, 5 °C can be viewed as a modification of the optimal regime that yields an even narrower macropore distribution with only a minor decrease in mesopore volume.

#### 2.3.2. Effect of Pulsed Pressure Changes

**Figure 6:**
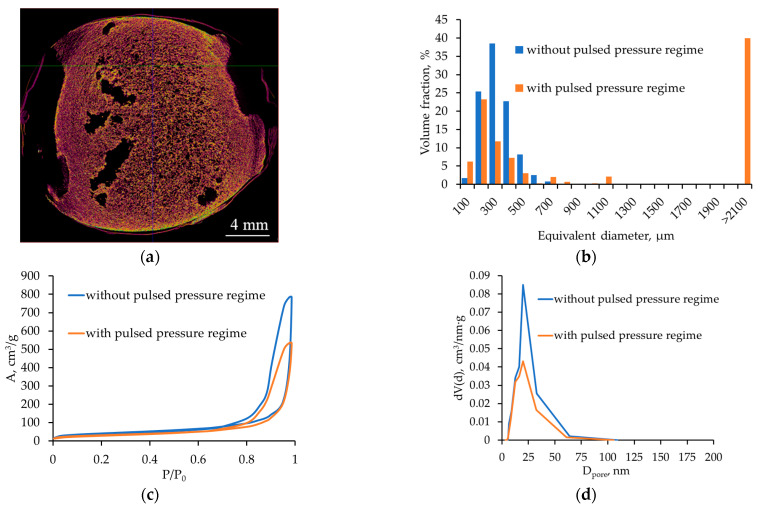
Micro-CT images and nitrogen sorption results for alginate aerogels (saturation pressure 100 bar; depressurization rate 0.05 bar/s; with and without pulsed pressure regime): (**a**,**b**) micro-CT and pore size distribution; (**c**,**d**) nitrogen adsorption–desorption isotherm and mesopore size distribution.

Figure 6 presents the micro-CT and nitrogen sorption results for alginate aerogels produced at a saturation pressure of 100 bar and a depressurization rate of 0.05 bar/s with pulsed pressure variation during foaming. The macropore size distribution was calculated from the micro-CT data.

Pulsed pressure variation during foaming at 100 bar and 0.05 bar/s switches the system from a quasi-stationary regime to a regime of periodic supersaturation changes, where short intervals of sharply increased dP/dt occur against the background of an overall slow depressurization. Micro-CT results (Figure 6a) show that pulsed pressure disrupts the spatial homogeneity of the structure: large cavities formed by bubble coalescence are clearly visible in the central region of the sample cross-section, whereas the peripheral zone retains a fine cellular texture similar to the initial optimal regime. In other words, pressure pulses generate large pores in regions where the local pressure drop is high and bubbles coarsen, while between pulses a fine-pored structure continues to form.

The macropore size histogram (Figure 6b) reveals that a substantial fraction of pores lies in the 100–300 µm range, but a pronounced secondary maximum appears at equivalent diameters > 2100 µm, corresponding to large macropores. Thus, pulsed pressure shifts the system from a narrow, nearly monodisperse distribution (100 bar, 0.05 bar/s) to a clearly bimodal structure: a population of small pores superimposed on a relatively small number of large but volumetrically significant cavities. This fundamentally alters the transport characteristics of the material: the fraction of convective transport pathways increases and structural homogeneity decreases.

Nitrogen sorption data show that the adsorption–desorption isotherm remains type IV, typical of mesoporous materials, but the rise at high relative pressures becomes steeper compared with the base regime of 100 bar, 0.05 bar/s, and the maximum in the mesopore size distribution shifts toward slightly larger diameters (~30–40 nm) and becomes broader.

The values of Pe and De, primarily determined by the 100 bar saturation pressure and the 0.05 bar/s average depressurization rate, remain within the region that would normally yield a homogeneous structure. However, during the pressure pulses the local depressurization rate increases sharply. Consequently, Pe and De temporarily shift into ranges characteristic of fast depressurization: diffusion cannot compensate for the abrupt pressure change, and the gel network cannot relax fast enough, which triggers bubble growth and coalescence and results in the formation of large macropores. Between pulses, the system returns to moderate Pe and low De, and fine porosity continues to develop. Thus, pulsed pressure produces alternating “favorable” and “coalescent” structural regimes, leading to a hierarchical macroporous structure. This may be promising for applications requiring both a high surface area and the presence of large macropores serving as main transport pathways, but is less attractive for mechanically robust scaffolds for cell culture.

#### 2.3.3. Effect of Surfactant Addition

**Figure 7:**
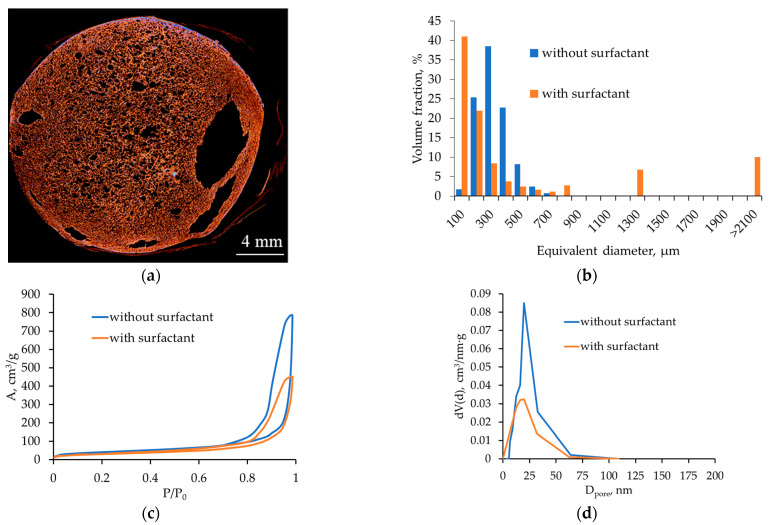
Micro-CT images and nitrogen sorption results for alginate aerogels (saturation pressure 100 bar; depressurization rate 0.05 bar/s; with and without surfactant): (**a**,**b**) micro-CT and pore size distribution; (**c**,**d**) nitrogen adsorption–desorption isotherm and mesopore size distribution.

Figure 7 shows the micro-CT and nitrogen sorption results for alginate aerogels produced at a saturation pressure of 100 bar and a depressurization rate of 0.05 bar/s with added surfactant.

Micro-CT results demonstrate that surfactant addition still yields a macroporous structure throughout the volume, but extended large macropores appear at the periphery and in local regions. In the pore size histogram (Figure 7b), most pores are in the 100–300 µm range, but there is a significant contribution from pores with equivalent diameters > 2100 µm, indicating a combination of intense nucleation of small pores with localized coalescence. Nitrogen sorption data show that the adsorption isotherm remains type IV, typical of mesoporous materials, and the mesopore size distribution has a maximum in the ~20–30 nm range.

Surfactant addition reduces interfacial tension, decreasing the critical nucleus radius and facilitating the formation of a large number of small bubbles. At the same time, interface stabilization limits their coalescence, increasing the fraction of small macropores and preserving a well-developed mesoporous network. Under the same Pe and De (i.e., with unchanged depressurization rate and network relaxation time), this leads to a local transition into a “coalescent” regime and the formation of large macropores observed in the micro-CT images. Regions near the material surface—where mass-transfer and stress gradients are more pronounced—are more prone to localized bubble coalescence and cell enlargement, resulting in larger pores.

Table 2 presents the results of nitrogen porosimetry, the total porosity values (P) calculated from the true and apparent densities, and the porosity determined by micro-CT (P_micro-CT_) for samples prepared using the modified methodology (5 °C, pulsed pressure variation, surfactant) as well as for the reference sample (100 bar, 0.5 bar/s). It should be noted that, in the micro-CT analysis, only pores with diameters in the range of 2–2100 µm were taken into account. Pores with larger diameters were excluded from the statistical analysis, as they were formed as a result of an uncontrolled pore coalescence process.

Modification of the fabrication methodology for materials with hierarchical porous structures—specifically, lowering the temperature, applying pulsed pressure during the foaming process, and adding a surfactant—leads to an increase in porosity within the 2–2100 µm range. This effect is associated with a shift in the macropore size distribution toward smaller pore sizes. Thus, the formation of smaller macropores is accompanied by an increase in porosity within the specified range.

Thus, the considered modifications—lowering the process temperature, applying pulsed pressure variation, and adding surfactant—significantly affect macropore formation and, to a lesser extent, the mesoporous structure.

## 3. Conclusions

Table 3 summarizes the effects of CO_2_-based foaming parameters (saturation pressure, depressurization rate, temperature, pulsed pressure variation) and surfactant addition on the hierarchical pore structure.

In summary, this work demonstrates that foaming in a CO_2_ environment is an effective tool for forming a hierarchical porous structure in alginate aerogels. The study confirms that the hierarchical porosity of materials produced by CO_2_ foaming is governed by two coupled parameters: saturation pressure and depressurization rate. Increasing pressure enhances CO_2_ solubility and decreases pH, thereby accelerating the release of dissociated Ca^2+^ ions and increasing the degree of network crosslinking before depressurization. This leads to a higher modulus and earlier formation of a solid network during bubble growth, which, at slow depressurization, allows the production of a fine macroporous structure and a mesoporous network with a narrow pore size distribution. Increasing the depressurization rate drives the system into a non-equilibrium regime characterized by higher Pe, local supersaturation, and larger pressure and concentration gradients, which cause pore wall rupture and coalescence. Thus, combining high pressure (100 bar) with slow depressurization (0.05 bar/s) yields a narrow macropore distribution (200–500 µm) and shifts mesopores into the 15–35 nm range.

Additionally, the effects of process temperature, pulsed pressure variation, and surfactant addition on hierarchical porosity were investigated. Their influence at fixed pressure and depressurization rate is consistent with the same fundamental principles. Lower temperature increases gel relaxation time and reduces the diffusion coefficient, effectively increasing De while maintaining Pe. Macropores become smaller and more homogeneous, and the mesopore distribution shifts toward smaller diameters with some broadening. During pulsed pressure variation, peak Pe values increase, leading to pore coarsening and coalescence. Surfactant addition lowers interfacial tension and, consequently, the critical nucleation radius, increasing the density of nuclei and stabilizing thin walls.

Overall, the results show that by varying pressure, depressurization rate, process temperature, and surfactant addition, it is possible to deliberately design the macropore hierarchy and tune the balance between structural homogeneity, mechanical stability, and transport properties for target scaffold applications.

Thus, the hierarchically porous materials produced with the addition of a surfactant, whose macropore volume is dominated by pores up to 100 µm, can promote efficient fibroblast adhesion and angiogenesis. Scaffolds obtained under reduced foaming temperatures and with pulsed pressure changes during foaming may be suitable for osteogenesis and chondrocyte growth.

The next stage of the research involves studying the developed materials with hierarchical porous structures using various cell lines, specifically assessing their cytotoxic, adhesive, and proliferative properties to evaluate their effectiveness in supporting cell growth for applications in tissue engineering.

Key limitations of CO_2_-induced alginate foaming/gelation stem from the fact that at scale, maintaining spatially uniform CO_2_ uptake, pH evolution, and nucleation/growth conditions across the entire scaffold is difficult because gas dissolution, diffusion, drainage, and heat/mass transfer become non-uniform with increasing length scales. Reproducibility is limited by stochastic bubble nucleation, time-dependent foam ageing (coarsening/coalescence and drainage) occurring concurrently with the moving gelation front, and formulation variability (polymer MW distribution, ionic strength, CaCO_3_ dispersion state). The use of Pluronic F-68 adds another reproducibility axis: poloxamers are known to show performance differences tied to physical structure and batch-to-batch composition, and their foam stability/surface tension behavior can shift with “impurities” or composition changes—factors that can propagate into pore-size distributions in foamed constructs.

Impact of process parameters on scaffold properties. Because pore architecture strongly governs cell infiltration, transport, and tissue formation, even modest shifts in pore size distribution and interconnectivity can materially change biological performance—and there is still no universal consensus on the “single optimal” pore size across tissues, reinforcing the need for tight control and reporting of distributions rather than averages alone. In CO_2_ foaming more generally, pore formation remains challenging to model, and pore size/distribution can be difficult to predict and precisely control.

## 4. Materials and Methods

### 4.1. Materials

Sodium alginate (Sigma-Aldrich, St. Louis, MO, USA) was used as the biopolymer for gel preparation. Pluronic F-68 (Gibco, Beijing, China) was used as a surfactant for macropore formation. Calcium carbonate (Ruskhim, Moscow, Russia) was used to carry out gelation and foaming in a CO_2_ environment. Isopropyl alcohol (IPA, Ruskhim, Moscow, Russia) was used as a solvent. Distilled water was prepared in the laboratory.

### 4.2. Methods

**Figure 8:**
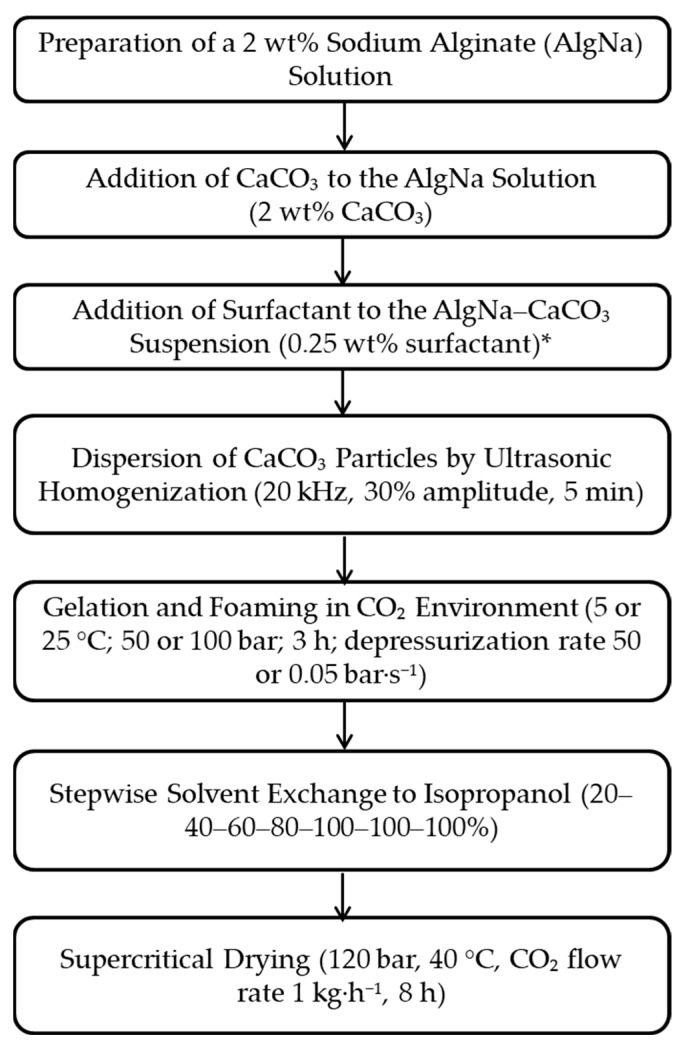
Process scheme for obtaining sodium alginate aerogels with a hierarchical porous structure (*—this stage is used only in Section 2.3.3).

A method was proposed to obtain sodium alginate-based aerogels while varying different parameters (Figure 8).

In this study, the effects of the following technological parameters on macropore formation were investigated: temperature (5 or 25 °C), pressure (50 or 100 bar), and depressurization rate (50 or 0.05 bar/s). In addition, an extra stage—pulsed pressure variation during gelation and foaming—was implemented. Pulsed pressure variation was defined as a decrease in pressure to 10 bar at a rate of 1.5 bar/s followed by repressurization to 100 bar, repeated every hour after the start of foaming. In total, three pressure pulses were applied. Furthermore, the effect of adding Pluronic F-68 (0.25 wt%) to the “sodium alginate–calcium carbonate” suspension immediately before gelation and foaming was studied. Parameters such as the concentration of the crosslinking agent (CaCO_3_), the operating conditions of the ultrasonic homogenization process, and the foaming time were investigated in earlier studies [38].

A detailed description of hierarchical structure formation in sodium alginate aerogels under different conditions is provided below.

#### 4.2.1. Alginate Dispersion Preparation

An aqueous sodium alginate solution with a concentration of 2 wt% was prepared according to the procedure. Calcium carbonate was added to the solution so that its concentration in the resulting suspension was 2 wt%. To ensure uniform CaCO_3_ distribution, the suspension was subjected to ultrasonic homogenization (Bandelin SONOPULS HD 4100) at 30% amplitude for 5 min. These amplitude and time values are sufficient to produce a stable suspension due to a reduction in CaCO_3_ particle size and uniform distribution throughout the volume. The operating frequency of the ultrasonic homogenizer was fixed at 20 kHz.

#### 4.2.2. CO_2_ Foaming Process

**Figure 9:**
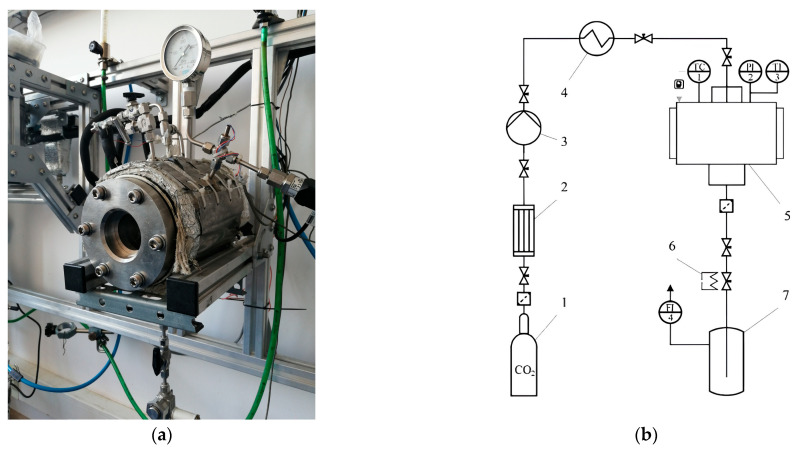
(**a**) Photograph and (**b**) process flow diagram of the setup for foaming and supercritical drying: 1—CO_2_ cylinder; 2—condenser; 3—pump; 4—heat exchanger; 5—250 mL high-pressure vessel; 6—heater; 7—separator; TC1—temperature controller; PI2—pressure gauge; TI3—thermocouple.

After homogenization, the suspensions were poured into Petri dishes (35 × 10 mm) and placed into a 250 mL high-pressure vessel (Figure 9). The suspension layer height was 5 mm. CO_2_ was introduced into the vessel. Carbon dioxide dissolved in the dispersion and created an acidic environment, thereby initiating the dissolution of calcium carbonate particles. Calcium cations crosslinked sodium alginate. After foaming, the gel samples were removed from the high-pressure vessel. The gels were cylindrical sponges with a height of 5 mm and a diameter of 35 mm.

#### 4.2.3. Supercritical CO_2_ Drying

For all samples, the solvent was stepwise replaced by isopropyl alcohol in preparation for supercritical drying. Stepwise replacement was carried out by sequentially increasing the solvent concentration in which the gels were immersed. The residence time at each step was 4 h, which was sufficient to reach equilibrium concentration within the gel.

Figure 9 shows the process flow diagram and the appearance of the setup used for supercritical drying.

Process parameters and duration were selected based on the literature [39] and were 120 bar and 40 °C, with a CO_2_ flow rate of 1 kg/h and a total drying time of 8 h.

### 4.3. Analytical Study

Samples were analyzed by micro-computed tomography using a SkyScan-1172 microtomograph (Bruker Corporation, Karlsruhe, Germany). The following visualization parameters were used: X-ray source voltage/current 25–50 kV/100–118 µA, nominal resolution 1.49 µm, and sample rotation step 0.2°.

The mesoporous structure was investigated by low-temperature nitrogen adsorption (77 K) using a NOVA 2200E surface area and porosity analyzer (Quantachrome Instruments Corp., Boynton Beach, FL, USA). Prior to analysis, samples were degassed at a pressure of 0.5 mmHg and a temperature of 313 K for 12 h to remove adsorbed moisture from the surface. The specific surface area was determined by the BET method, while mesopore size distribution and volume were calculated by the BJH method.

Measurements of the true density were carried out using the method of helium pycnometry. The true density measurements were performed on an Anton Paar Ultrapyc 5000 automatic helium pycnometer. The values of the true and apparent density make it possible to determine the porosity of the material using the Formula (6):

(6) $$P=\left(1-\frac{\rho_{b}}{\rho_{tr}}\right)\cdot 100\%,$$

where *P*—porosity, %; $\rho_{b}$—bulk density, g/cm^3^; $\rho_{tr}$—true density, g/cm^3^

## Figures and Tables

**Table 1:** Nitrogen sorption results.

Parameters	S_BET_, m^2^/g	V_BJH_, cm^3^/g
100 bar and 50 bar/s	206	2.0
100 bar and 0.05 bar/s	148	1.3
50 bar and 50 bar/s	127	0.9
50 bar and 0.05 bar/s	117	0.8

**Table 2:** Nitrogen sorption and micro-CT results.

Parameters	S_BET_, m^2^/g	P, %	P_micro-CT_, % (2–2100 µm)
100 bar and 0.05 bar/s (comparison sample)	148	95 ± 2%	6.32 ± 2%
Low process temperature (5 °C)	112	95 ± 2%	16.48 ± 2%
Pulsed pressure regime	107	95 ± 2%	20.64 ± 2%
Adding surfactant	107	95 ± 2%	20.93 ± 2%

**Table 3:** Summary of the effects of CO_2_-foaming process parameters on hierarchical porosity.

Parameter	Mechanism	Macroporous Structure	Mesoporous Structure
Pressure (50 → 100 bar)	↑ CO_2_ solubility → ↓ pH → ↑ Ca^2+^ release → ↑ network stiffness at the moment of depressurization; ↑ supersaturation	Slow depressurization: pores (200–500 µm) with narrow size distribution. Fast depressurization: ↑ coalescence, formation of large pores (>2100 µm)	At 100 bar and slow depressurization: narrow distribution (20–35 nm). At fast depressurization: broader distribution (30–60 nm)
Depressurization rate (50 → 0.05 bar/s)	↑ depressurization time → ↑ De, ↓ Pe (quasi-equilibrium depressurization, ↓ coalescence)	Narrower pore size distribution and ↓ mean diameter	Narrow mesopore size distribution combined with high surface area
Temperature (25 → 5 °C)	↑ CO_2_ solubility; ↑ viscoelastic properties and relaxation time; ↓ diffusion coefficient	More homogeneous porous structure	Shift of distribution peak toward smaller diameters (12–25 nm)
Pulsed pressure variation	Repeated ΔP peaks: local ↑ Pe, directed flows and stress fields	Local coalescence, pores 100–300 µm; formation of large pores (>2100 µm)	No significant effect on mesostructure
Surfactant addition	↓ interfacial tension → ↓ critical radius; stabilization of thin walls	Fine macroporous structure (100–300 µm) with peripheral coalescence	Shift of distribution peak toward smaller diameters (12–25 nm)

↑ means an increase, ↓ means a decrease.

## Data Availability

The original contributions presented in this study are included in the article. Further inquiries can be directed to the corresponding authors.

## References

[1] Thakare N.R., Hazarika S. (2025). Eco-friendly biopolymers and their biomedical applications: A review. Int. J. Biol. Macromol..

[2] Shu J., McClements D.J., Luo S., Liu C., Ye J. (2025). Advances of biopolymer-based emulsion gels: Fabrication, design, and application. Trends Food Sci. Technol..

[3] Sha Y., Li S., Li X., He M., Wu Y., Gao Y., Hu J., Mai X. (2025). Aerogels of Cellulose Nanofibers@Metal-Organic Frameworks for Carbon Dioxide Capture. Eng. Sci..

[4] Dehghani F., Annabi N. (2011). Engineering porous scaffolds using gas-based techniques. Curr. Opin. Biotechnol..

[5] Menshutina N.V., Uvarova A.A., Mochalova M.S., Lovskaya D.D., Tsygankov P.Y., Gurina O.I., Zubkov E.A., Abramova O.V. (2023). Biopolymer Aerogels as Nasal Drug Delivery Systems. Russ. J. Phys. Chem. B.

[6] Jeon O., Powell C., Solorio L., Krebs M., Alsberg E. (2011). Affinity-based growth factor delivery using biodegradable, photocrosslinked heparin-alginate hydrogels. J. Control. Release.

[7] Karageorgiou V., Kaplan D. (2005). Porosity of 3D biomaterial scaffolds and osteogenesis. Biomaterials.

[8] Han Y., Lian M., Wu Q., Qiao Z., Sun B., Dai K. (2021). Effect of Pore Size on Cell Behavior Using Melt Electrowritten Scaffolds. Front. Bioeng. Biotechnol..

[9] Djemaa I., Andrieux S., Auguste S., Jacomine L., Tarnowska M., Drenckhan-Andreatta W. (2022). One-Step Generation of Alginate-Based Hydrogel Foams Using CO_2_ for Simultaneous Foaming and Gelation. Gels.

[10] Deepika D., Jagadeeshbabu J.P. (2019). Sacrificial Polystyrene Template Assisted Synthesis of Tunable Pore Size Hollow Core-Shell Silica Nanoparticles (HCSNs) for Drug Delivery Application. AIP Conf. Proc..

[11] Santos-Rosales V., Ardao I., Alvarez-Lorenzo C., Ribeiro N., Oliveira A.L., García-González C.A. (2019). Sterile and Dual-Porous Aerogels Scaffolds Obtained through a Multistep Supercritical CO_2_-Based Approach. Molecules.

[12] Santos-Rosales V., Alvarez-Rivera G., Hillgärtner M., Cifuentes A., Itskov M., García-González C.A., Rege A. (2020). Stability Studies of Starch Aerogel Formulations for Biomedical Applications. Biomacromolecules.

[13] Qiao M., Yang X., Zhu Y., Guerin G., Zhang S. (2020). Ultralight Aerogels with Hierarchical Porous Structures Prepared from Cellulose Nanocrystal Stabilized Pickering High Internal Phase Emulsions. Langmuir.

[14] Ceccaldi C., Bushkalova R., Cussac D., Duployer B., Tenailleau C., Bourin P., Parini A., Sallerin B., Girod Fullana S. (2017). Elaboration and Evaluation of Alginate Foam Scaffolds for Soft Tissue Engineering. Int. J. Pharm..

[15] Catanzano O., Soriente A., La Gaa A., Cammarota M., Ricci G., Fasolino I., Schiraldi C., Ambrosio L., Malinconico M., Laurienzo P. (2018). Macroporous Alginate Foams Crosslinked with Strontium for Bone Tissue Engineering. Carbohydr. Polym..

[16] Wang B., Li D., Tang M., Ma H., Gui Y., Tian X., Quan F., Song X., Xia Y. (2018). Alginate-Based Hierarchical Porous Carbon Aerogel for High-Performance Supercapacitors. J. Alloys Compd..

[17] Menshutina N., Fedotova O., Abramov A., Golubev E., Sulkhanov Y., Tsygankov P. (2024). Processes of Obtaining Nanostructured Materialswith a Hierarchical Porous Structure on the Example of Alginate Aerogels. Gels.

[18] Zhou Y., Tian Y., Zhang M. (2024). Technical development and application of supercritical CO_2_ foaming technology in PCL foam production. Sci. Rep..

[19] Valor D., Montes A., Monteiro M., García-Casas I., Pereyra C., Martínez de la Ossa E. (2021). Determining the Optimal Conditions for the Production by Supercritical CO_2_ of Biodegradable PLGA Foams for the Controlled Release of Rutin as a Medical Treatment. Polymers.

[20] Fanovich M.A., Di Maio E., Salerno A. (2023). Current Trend and New Opportunities for Multifunctional Bio-Scaffold Fabrication via High-Pressure Foaming. J. Funct. Biomater..

[21] Kurki A., Paakinaho K., Hannula M., Hyttinen J., Miettinen S., Sartoneva R. (2023). Ascorbic Acid 2-Phosphate-Releasing Supercritical Carbon Dioxide-Foamed Poly(L-Lactide-Co-epsilon-Caprolactone) Scaffolds Support Urothelial Cell Growth and Enhance Human Adipose-Derived Stromal Cell Proliferation and Collagen Production. J. Tissue Eng. Regen. Med..

[22] Souto-Lopes M., Fernandes M.H., Monteiro F.J., Salgado C.L. (2023). Bioengineering Composite Aerogel-Based Scaffolds That Influence Porous Microstructure, Mechanical Properties and In Vivo Regeneration for Bone Tissue Application. Materials.

[23] Florea-Spiroiu M., Bala D., Balan A., Nichita C., Stamatin I. (2012). Alginate matrices prepared in sub and supercritical CO_2_. Dig. J. Nanomater. Biostruct..

[24] Namiyut A.Y. (1991). Solubility of Gases in Water: Reference Handbook.

[25] Diamond L., Akinfiev N. (2003). Solubility of CO_2_ in water from −1.5 to 100 °C and from 0.1 to 100 MPa: Evaluation of literature data and thermodynamic modelling. Fluid Phase Equilibria.

[26] Winter H.H., Chambon F. (1986). Analysis of Linear Viscoelasticity of a Crosslinking Polymer at the Gel Point. *J.* Rheol..

[27] Leung S.N., Wong A., Guo Q., Park C.B., Zong J.H. (2009). Change in the Critical Nucleation Radius and Its Impact on Cell Stability during Polymeric Foaming Processes. Chem. Eng. Sci..

[28] Shafi M.A., Flumerfelt R.W. (1997). Initial Bubble Growth in Polymer Foam Processes. Chem. Eng. Sci..

[29] Venerus D.C. (2003). Modeling Diffusion-Induced Bubble Growth in Polymer Liquids. Cell. Polym..

[30] Breuer R. (2021). Modeling Flow and Cell Formation in Foam Sheet Extrusion of Polystyrene with CO_2_ and Co-Blowing Agents. Part II: Process Model. Polym. Eng. Sci..

[31] Venerus D.C., Yala N., Bernstein B. (1998). Analysis of Diffusion-Induced Bubble Growth in Viscoelastic Liquids. J. Non-Newton. Fluid Mech..

[32] Tammaro D., Villone M.M., D’Avino G., Maffettone P.L. (2022). An Experimental and Numerical Investigation on Bubble Growth in Polymeric Foams. Entropy.

[33] Poole R.J. (2012). The Deborah and Weissenberg Numbers. Rheol. Bull..

[34] Lombardi L., Tammaro D. (2021). Effect of Polymer Swell in Extrusion Foaming of Low-Density Polyethylene. Phys. Fluids.

[35] Malektaj H., Drozdov A.D., deClaville Christiansen J. (2023). Mechanical Properties of Alginate Hydrogels Cross-Linked with Multivalent Cations. Polymers.

[36] Abramov A.A., Okisheva M.K., Tsygankov P.Y., Menshutina N.V. (2023). Development of “Ink” for Extrusion Methods of 3D Printing with Viscous Materials. Russ. J. Gen. Chem..

[37] Everitt S.L., Harlen O.G., Wilson H.J. (2006). Bubble Growth in a Two-Dimensional Viscoelastic Foam. J. Non-Newton. Fluid Mech..

[38] Abramov A.A., Tsygankov P.Y., Golubev E.V., Menshutina N.V. (2024). Formation of a hierarchical structure in aerogels based on sodium alginate using the foaming process in a carbon dioxide environment. Mod. High Tech. Reg. App..

[39] Menshutina N., Tsygankov P., Khudeev I., Lebedev A. (2022). Methods of intensification of supercritical drying for obtaining aerogels. Dry. Technol..

